# Solution-Based Deposition of Transparent Eu-Doped Titanium Oxide Thin Films for Potential Security Labeling and UV Screening

**DOI:** 10.3390/nano10061132

**Published:** 2020-06-08

**Authors:** Anara Molkenova, Laura Khamkhash, Ainur Zhussupbekova, Kuanysh Zhussupbekov, Sagyntay Sarsenov, Izumi Taniguchi, Igor V. Shvets, Timur Sh. Atabaev

**Affiliations:** 1 Department of Chemistry, Nazarbayev University, Nur-Sultan 010000, Kazakhstan; sagyntay.sarsenov@nu.edu.kz; 2 Core Facilities, Nazarbayev University, Nur-Sultan 010000, Kazakhstan; laura.khamkhash@nu.edu.kz; 3 School of Physics and Centre for Research on Adaptive Nanostructures and Nanodevices (CRANN), Trinity College Dublin, Dublin, Ireland; zhussupa@tcd.ie (A.Z.); zhussupk@tcd.ie (K.Z.); igor.chvets@tcd.ie (I.V.S.); 4 Department of Chemical Science and Engineering, Tokyo Institute of Technology, Tokyo 152-8550, Japan; taniguchi.i.aa@m.titech.ac.jp

**Keywords:** transparent thin film, titanium oxide, europium (III) doping, security labeling, UV screening

## Abstract

Transparent titanium oxide thin films attract enormous attention from the scientific community because of their prominent properties, such as low-cost, chemical stability, and optical transparency in the visible region. In this study, we developed an easy and scalable solution-based process for the deposition of transparent TiOx thin films on glass substrates. We showed that the proposed method is also suitable for the fabrication of metal-doped TiOx thin films. As proof-of-the-concept, europium Eu(III) ions were introduced into TiOx film. A photoluminescence (PL) study revealed that Eu-doped TiOx thin films showed strong red luminescence associated with ^5^D_0_→^7^F_j_ relaxation transitions in Eu (III). We found that prepared TiOx thin films significantly reduce the transmittance of destructive UV radiation; a feature that can be useful for the protection of photovoltaic devices. In addition, transparent and luminescent TiOx thin films can be utilized for potential security labeling.

## 1. Introduction

Titanium oxides with a general formula TiOx (titanium oxide) are versatile and low-cost materials suitable for numerous applications. For example, well-known titanium dioxide TiO_2_ is used as a white pigment in sunscreen and enamels [1], as a food additive [2], and in photocatalytic reactions [3,4]. Semitransparent thin films made of TiO_2_ nanoparticles are used as an electron transporting material in photovoltaic devices [5,6]. TiO_2_ thin films with high transparency in the visible range can be employed as UV-protective coatings for photovoltaic devices [7,8]. To date, some sophisticated methods, such as chemical vapor deposition [7], spray pyrolysis [8], pulsed laser deposition [9], and RF magnetron sputtering [10] are widely employed for the deposition of titanium oxide thin films. However, these methods either require vacuum conditions and high voltages (pulsed laser deposition, magnetron sputtering) or high temperatures and environmental control (chemical vapor deposition, spray pyrolysis), that are costly and energy-consuming. In this regard, spin coating method can be considered as a low-cost and user-friendly method. For example, TiO_2_ nanoparticles based thin films are commonly reported in the literature [11,12]. However, the rough morphology, semitransparent nature, and cracks formed in these thin films hinder their practical applications.

It is well-known that the doping process can significantly change the physicochemical properties of titanium oxide. For example, one can easily alter the bandgap, recombination rate of electron-hole pairs, conductivity and optical properties of prepared films [13]. Among different ions, europium element is a well-studied activator for the preparation of red-emitting optical materials [14,15]. L. Song and coauthors [16] showed that Eu-doped TiO_2_ nanotubes exhibited high photocatalytic activity under visible light illumination compared to a commercial P25 TiO_2_ powder. Another report suggested that Eu-doped TiO_2_ thin films can improve the performance of organic solar cells [17]. It was shown that Eu-doping improves the electron transport properties, reduces the electron-hole recombination rate, and lowers the bandgap values of TiO_2_ [16,17]. Therefore, the development of an easy and low-cost deposition method of transparent undoped/doped titanium oxide thin films is important from scientific and technological points of view.

In this study, we presented a novel and simple solution-based deposition of transparent TiOx thin films on glass slides, using spin coating at ambient conditions. Fabrication simplicity and excellent reproducibility highlight the potential applicability of the proposed method for the generation of functional coatings for security labeling, UV screening, photovoltaic devices, etc.

## 2. Materials and Methods

### 2.1. Film Deposition

Titanium isopropoxide TIP (>97.0%), anhydrous 1-butanol (99.8%), absolute ethanol (≥99.8%), and EuCl_3_ × 6H_2_O (99.9%) were purchased from Merck & Co. (Kenilworth, NJ, USA) and used as received. A precursor solution for TiOx film was prepared by mixing ethanol (0.5 mL), 1-butanol (1 mL), and 100 µL of TIP. For Eu-doped TiOx film, 10 mg of europium salt was firstly dissolved in 0.5 mL of ethanol and then mixed with 1 mL of 1-butanol and 100 µL of TIP. Later on, the precursor solution was spin-coated on clean glass slides (20 × 15 mm) at 500 rpm (5 s), followed by 2000 rpm (15 s). All experiments were repeated five times to ensure the reproducibility of the results. Obtained thin films were naturally dried in ambient conditions for 2 h and then annealed at 500 °C (heating rate 5 °C/min) for 1 h. Annealed thin films were used for further testing.

### 2.2. Characterization

X-ray diffraction (XRD) measurements were performed using a SmartLab X-ray Diffractometer (Rigaku Corp., Tokyo, Japan) with a Cu Kα radiation source. X-ray photoelectron spectroscopy (XPS) was performed in an Omicron MultiProbe XPS (Scienta Omicron Inc.,Uppsala, Sweden) using a monochromized Al Kα source (XM 1000, 1486.7 eV). The instruments’ base pressure was 5 × 10^−11^ mbar and the instrumental resolution was 0.6 eV. Samples were attached to the sample holder by a copper tape. A charge neutralizer was used during the measurements. Atomic force microscope (AFM, SmartSPM 1000, AIST-NT Inc., Novato, CA, USA) was used to obtain topographic images of film surfaces. UV-Vis light transmittance measurements were conducted using a Genesys 50 UV-Visible spectrophotometer (Thermo Fisher Scientific Inc., Waltham, MA, USA). The optical properties of films were examined using a fluorescence spectrophotometer (Agilent Cary Eclipse, Agilent Technologies Inc., Santa Clara, CA, USA).

## 3. Results and Discussion

**Figure 1:**
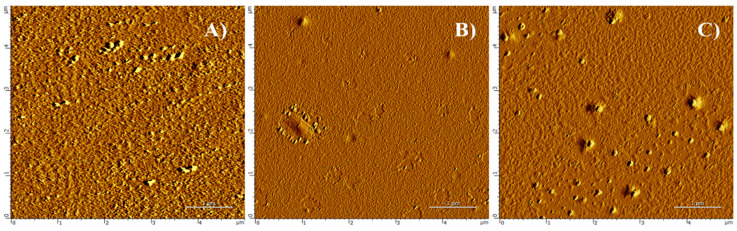
Atomic force microscope (AFM) 2D images of (**A**) bare glass slide, (**B**) undoped TiOx, and (**C**) Eu-doped TiOx thin films.

**Figure 2:**
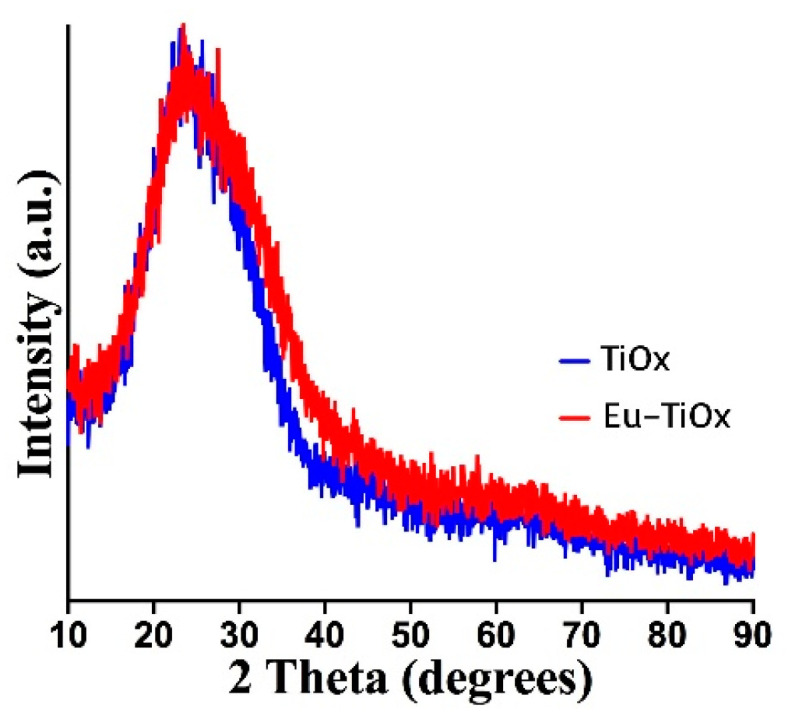
XRD patterns of undoped and Eu-doped TiOx thin films.

It is interesting to note that this methodology can be used to fabricate metal-doped TiOx thin films. Herein, we introduced europium salt to achieve a red-emitting luminescent thin film. AFM was utilized to examine the surface topography and surface roughness of the prepared films. Figure 1 shows corresponding 2D images of the control sample (bare glass slide), undoped TiOx film, and Eu-doped TiOx film captured on an area of 5 × 5 µm. One can easily observe that the surface of the control sample (Figure 1a) becomes less rough after the deposition of TiOx thin films (Figure 1b,c). The average surface roughnesses (Ra) estimated by AFM were found to be 96.1, 54.7, and 66.3 nm for control, undoped and Eu-doped TiOx, respectively. An X-ray reflectivity (XRR) analysis revealed that the thickness of undoped TiOx and Eu-doped TiOx thin films were ~33 nm and ~37 nm respectively. Figure 2 shows XRD patterns of undoped and Eu-doped TiOx thin films. In both cases, one can observe the formation of amorphous TiOx phase.

**Figure 3:**
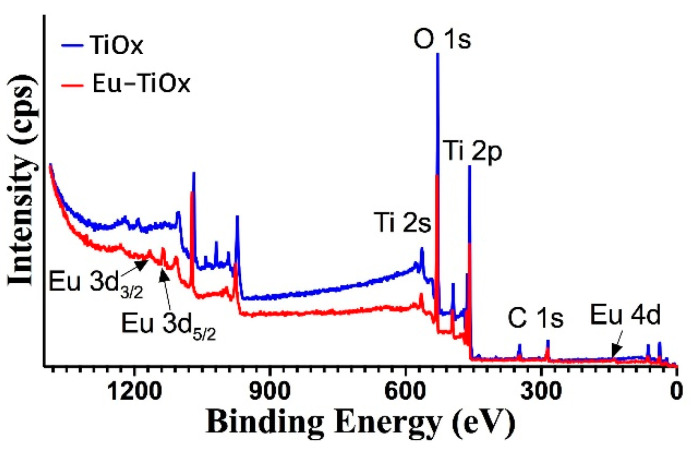
XPS surface analysis of undoped and Eu-doped TiOx thin films.

Figure 3 presents the wide-scan XPS survey spectra of TiOx and Eu-doped TiOx thin films. The survey indicates the presence of titanium, oxygen, and carbon in both films. The presence of C 1s can be attributed to the carbon contamination caused by exposure of the film surface to the atmosphere [18]. All energetic positions are corrected with respect to C1s (284.8 eV). Casa XPS software was employed to quantify measured regions. Components for fitting were selected from the elemental library according to peak position, with Gaussian/Lorentzian line-shapes and Shirley background.

**Figure 4:**
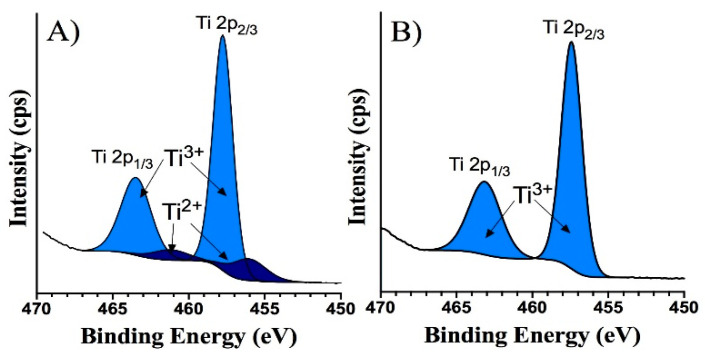
Narrow scan XPS spectra of Ti2p region in (**A**) undoped and (**B**) Eu-doped TiOx thin films.

**Figure 5:**
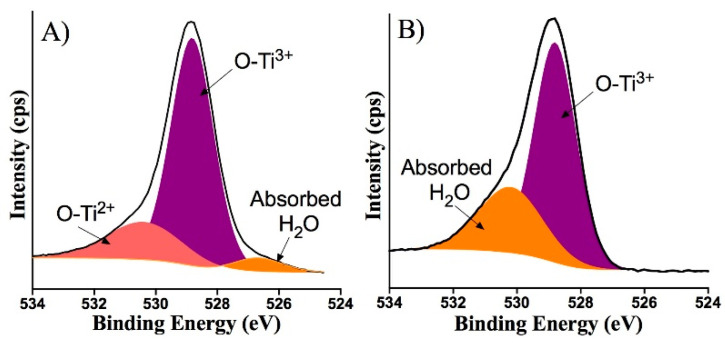
Narrow scan XPS spectra of O 1s region in (**A**) undoped and (**B**) Eu-doped TiOx thin films.

Figure 4 shows the narrow scan spectra of Ti 2p for both undoped and doped films. One can see that the Ti 2p spectrum of undoped film is composed of spin-orbit split peaks at binding energies of 457.75 eV (Ti 2p_3/2_) and 463.46eV (Ti 2p_1/2_). These binding energies are consistent with the Ti^3+^ state [19]. In addition, there was a small shoulder at the binding energy around 456 eV, indicating the presence of Ti^2+^ oxidation state. These results suggest that the experimental conditions yielded the TiOx film with the mixed oxidation states of titanium (Ti^3+/^Ti^2+^). The incorporation of Eu ions into a TiOx matrix led to a chemical shift in the Ti 2p_3/2_ and Ti 2p_1/2_ peaks to 457.4 eV and 463.16 eV, respectively. This shift in binding energies indicates an influence of Eu ion addition on the electronic state of titanium. However, these binding energies of the shifted Ti 2p peaks in Eu-doped TiOx film could still be assigned to the Ti^3+^ state [20]. Figure 5 demonstrates the narrow scan of O 1s, for both undoped and doped samples. It is clear that an additional oxygen state is present in the undoped sample (apart from main lattice oxygen peak and peak related to surface contamination), advocating the presence of the additional Ti state, as mentioned before.

**Figure 6:**
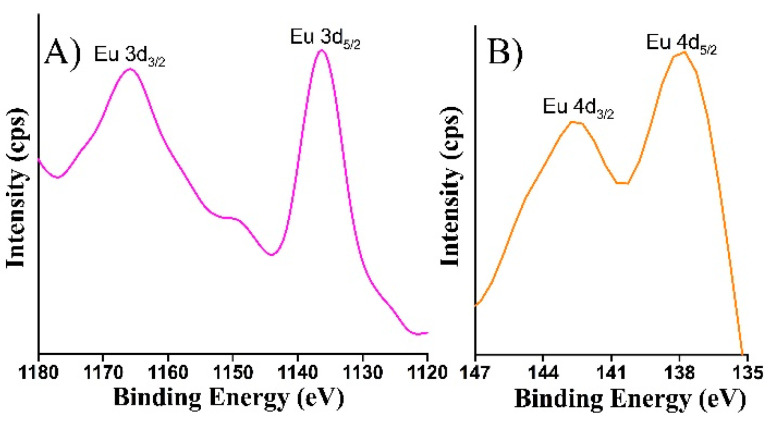
Narrow scan XPS spectra of (**A**) Eu 3d and (**B**) Eu 4d region in Eu-doped TiOx thin film.

Furthermore, careful examination of the wide XPS survey spectrum of the Eu-doped TiOx thin film (Figure 3) revealed that the sample contains Eu 3d and Eu 4d peaks. As shown in Figure 6, the narrow scan of Eu 3d spectrum recorded from the Eu-doped TiOx thin film is composed of spin-orbit split peaks at binding energies of 1136.7 eV (Eu 3d_5/2_) and 1165.7 eV (Eu 3d_3/2_). In addition, the narrow scan Eu 4d spectrum is composed of spin−orbit peaks at binding energies of 142.8 eV (Eu 4d_3/2_) and 137.7 eV (Eu 4d_5/2_). These binding energies are highly consistent with Eu^3+^ state [21]. According to XPS analysis, the elemental concentration was found to be 24.3% (Ti), 75.06% (O) and 0.54% (Eu).

**Figure 7:**
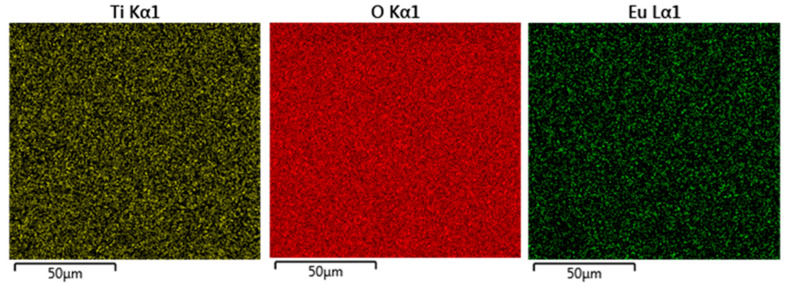
EDX elemental mapping of Eu-doped TiOx thin film.

Figure 7 shows the energy dispersive X-ray (EDX) elemental mapping of the Eu-doped TiOx film. One can easily observe that Eu ions are uniformly detected in the selected area. However, the distribution uniformity of other metal dopants should be verified separately.

**Figure 8:**
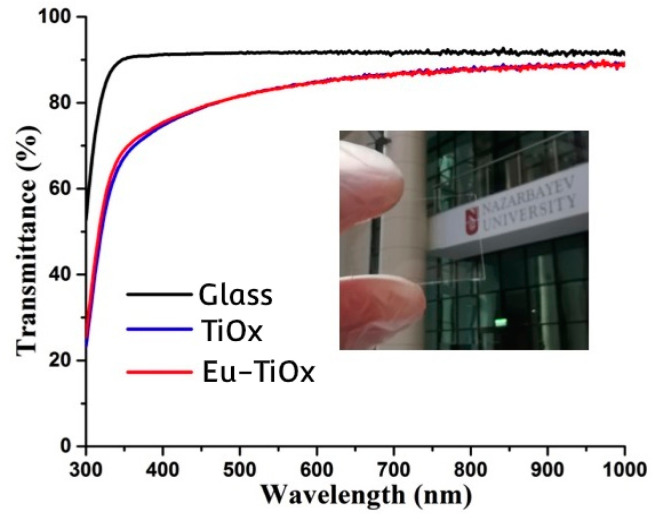
UV-Vis spectrum of a bare glass slide, undoped and Eu-doped TiOx films. Inset is a digital image of Eu-doped TiOx film deposited on a glass slide.

Figure 8 shows the transmittance of undoped and Eu-doped TiOx films deposited on glass substrates. The transmittance of a bare glass slide was also provided for reference. One can see that the transmittance of undoped TiOx and Eu-doped TiOx films were similar and slightly decreased compared to a bare glass slide. In particular, the transmittance in the visible range (fixed at 550 nm) was 91.7% (bare glass slide), 83.5% (undoped film), and 83.3% (Eu-doped film). However, a significant decrease of transmittance in the UV region suggested the potential applicability of prepared TiOx films for UV screening [7,8]. Figure 8, inset, shows that Eu-doped TiOx film is visually transparent.

**Figure 9:**
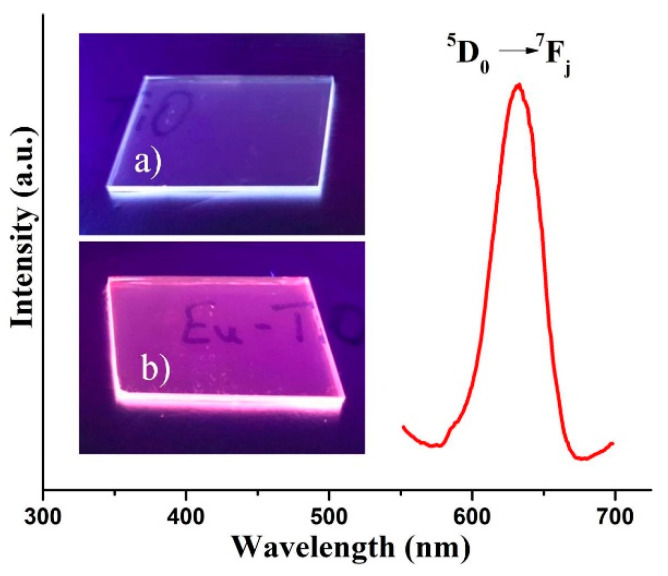
Room temperature emission of Eu-doped TiOx film. Inset are digital pictures of a) undoped and b) Eu-doped TiOx films under the excitation of commercially available UV lamp (λ_exc._ = 302 nm).

Figure 9 shows a PL emission of Eu-doped TiOx films (λ_exc._ = 310 nm), measured at room temperature RT in the range of 550–700 nm. A well-resolved broad emission peak, with an emission maximum at 633 nm, was detected. This emission is associated with radiative ^5^D_0_→ ^7^F_j_ (j = 0, 1, 2, and 3) transitions within Eu^3+^ ions [14,22]. However, at RT, these transitions are overlapped and detected as one broad peak in the red region. Inset of Figure 9 shows digital images of undoped and Eu-doped TiOx films under the excitation of a commercially available UV lamp (λ_exc._ = 302 nm). Red emission from a transparent Eu-doped TiOx film can be visually observed by a naked eye, making it suitable for the potential security labeling of valuable goods and photovoltaic applications [17].

## 4. Conclusions

In summary, we introduced a novel solution-based method for the deposition of transparent TiOx thin films using a spin coating method. It was found that nearly uniform thin film with a thickness of 30–40 nm can be formed. We also showed that the same method can be employed for the fabrication of metal-doped TiOx films. In particular, characteristic eye-visible red emission associated with Eu(III) ^5^D_0_→^7^F_j_ radiative transitions was observed for Eu-doped TiOx film. Fabrication simplicity, chemical stability, and excellent luminescent properties make Eu-doped TiOx films promising for UV screening, security labeling, and photovoltaic applications.
